# Histone demethylase JMJD6 regulates cellular migration and proliferation in adipose-derived mesenchymal stem cells

**DOI:** 10.1186/s13287-018-0949-3

**Published:** 2018-08-09

**Authors:** Chongyang Shen, Qingli Quan, Chuan Yang, Yueqiang Wen, Hong Li

**Affiliations:** 1 Key Laboratory of Obstetric and Gynecologic and Pediatric Diseases and Birth Defects of the Ministry of Education, West China Second University Hospital, Sichuan University, Chengdu, People’s Republic of China; 2 Basic Medicine School, Chengdu University of Traditional Chinese Medicine, Chengdu, People’s Republic of China; 3 West China Hospital, Sichuan University, Chengdu, People’s Republic of China

**Keywords:** Histone demethylase, JMJD6, ADSCs, Migration, Proliferation

## Abstract

**Background:**

Adipose-derived mesenchymal stem cells (ADSCs) have been extensively explored as a promising therapeutic agent due to their differentiation, proliferation and migration abilities. The epigenetic mechanisms that regulate the fate of mesenchymal stem cells (MSCs) have been described in detail. However, the epigenetic modulation of ADSCs proliferation and migration is poorly understood.

**Methods:**

The present study examined histone demethylases roles and expression by RT-PCR, as well as through siRNA screening and ChIP-qPCR assay. Cellular proliferation and migration assays were employed in shRNA-mediated JMJD6 knockdown and control ADSCs. PDE1C inhibition studies were conducted to confirm its role in JMJD6-mediated epigenetic regulation of ADSCs.

**Results:**

The data demonstrate that the histone demethylase JMJD6 plays a critical role in regulating the proliferation and migration of ADSCs by removing H4R3me2a at the promoter regions of PDEC1 and suppressing PDEC1 expression. Importantly, the depletion of JMJD6 in ADSCs significantly increased cellular proliferation and motility, which was associated with increases in PDE1C expression and decreases in the levels of both cAMP and cGMP. The increase in proliferation and migration was reversed by treatment with a PDE1C inhibitor, suggesting that JMJD6 attenuates the proliferation and migration of ADSCs as an epigenetic regulator and PDE1C partially contributes to the JMJD6-mediated regulation.

**Conclusions:**

Taken together, our results indicate for the first time that JMJD6 plays an important role in the regulation of ADSCs proliferation and migration through the modulation of PDE1C expression.

**Electronic supplementary material:**

The online version of this article (10.1186/s13287-018-0949-3) contains supplementary material, which is available to authorized users.

## Background

Mesenchymal stem cells (MSCs) can be isolated from a variety of tissues and used for cell-based therapies [1, 2]. The unique adipose-derived mesenchymal stem cells (ADSCs) exhibit similar properties to bone marrow mesenchymal stem cells (BM-MSCs) including proliferative potential and broad differentiation plasticity. Recently, ADSCs have been used in several stem cells therapy approaches. However, a lack of specific accumulation of ADSCs in injured tissues and limited cell proliferation in repeated subcultures in vitro reduces their therapeutic efficacy [3]. ADSCs capacity for differentiation, proliferation, and migration is critical to their efficacy in clinical applications. It has been suggested that MSCs can increase their proliferation rate and migratory activity during certain pathological conditions caused by changes in their microenvironment during tissue repair [4, 5].

It is universally accepted that epigenetic regulation is inherited and governs cell-specific gene expression without changes in the DNA sequence. This is mediated by chromatin remodeling, DNA methylation, non-coding RNAs, and histone modifications, which are elaborately controlled, and influence phenotypic commitment. Several recent studies reported that histone demethylases play a pivotal role in determining the fate of stem cells in many processes, including embryonic development, stem cell self-renewal and MSCs differentiation, by removing methyl groups from various histones that bind to cell-specific genes involved in differentiation toward a specific cell lineage [6–10]. Lee and Ye identified KDM4B and KDM6B as Jumonji-C domain containing histone demethylases that have epigenetic regulatory functions in the osteogenic differentiation of MSCs via the removal of H3K9me3 and H3K27me3 [8, 11]. Liu et al. demonstrated that the histone demethylase KDM6B enhances osteogenic differentiation and the anti-inflammatory properties of MSCs by increasing histone K27 methylation at the IGFBP5 gene promoter [9]. In addition, it has been shown that the histone demethylase LSD1 facilitates adipocyte differentiation by demethylating H3K4 at the promoter sites of Wnt signaling pathway genes [12].

The directed migration of ADSCs is critical for clinical applications, and the precise mechanisms underlying ADSCs migrations to injury sites have been thoroughly described. These mechanisms can be categorized as: (1) chemotactic cytokines that stimulate cell recruitment (MCP-1, RANTES, SDF-1, etc.) [13, 14]; (2) integrin receptors that promote cell migration (CD44, CD29, CD47, etc.) [15, 16]; (3) chemokine receptors that regulate cell mobilization (CCR1, CCR2, CXCR4, etc.) [3, 17]; and (4) growth factors that exert a strong chemotactic stimulus (PDGF, bFGF, HGF, etc.) [18, 19]; However, there may be additional mechanisms that mediate ADSCs migration. The main factors that drive ADSCs migration are well-characterized. In this study, we specifically investigated the epigenetic regulatory mechanisms of these signaling networks. To further identify specific histone demethylases that are associated with ADSCs migration, we systemically profiled the histone demethylases expression in ADSCs and conducted an RNA interference screening. We identified the histone demethylase Jumonji C domain-containing protein 6 (JMJD6) as a key epigenetic regulator that impairs ADSCs migration. JMJD6, was first described by Chang et al. [20], and is capable of removing methyl groups from histone dimethyl symmetric H4R3 (H4R3me2s) and histone dimethyl asymmetric H4R3 (H4R3me2a) [21, 22], acting as a transcriptional regulator. The H4R3me2s modification is associated with transcriptional repression and H4R3me2a is correlated with transcriptional activation [23]. In our study, knockdown of JMJD6 expression by shRNA increased the migration and proliferation potential of ADSCs in vitro by decreasing the demethylation of H4R3me2a at the phosphodiesterase 1C (PDE1C) promoters.

## Methods

### Cell culture and viral infection

Human adipose-derived mesenchymal stem cells (ADSCs) was purchased from Sichuan Mesenchymal stem cell bank and cultured in α-MEM (Invitrogen, Carlsbad, CA, USA) supplemented with 10% heat-inactivated FBS (Gibco, Carlsbad, CA, USA), 2 mM L-glutamine (Gibco), 100 U/ml penicillin (Gibco), and 100 μg/ml streptomycin (Gibco), at 37 °C in a 5% CO2 incubator (Sanyo, Osaka, Japan). Cells were passaged with trypsin at 80% confluence, growth medium was changed every 3 days. To generate stable knockdown JMJD6 cell lines, JMJD6 shRNAs were cloned into PLKO.1 vector (Addgene, Cambridge, MA, USA) and packaged in 293 T cells as described previously [7, 24], The sequences of shRNAs are showed in (Additional file 1: Table S1). To generate rescue cell lines, the sequence encoding human JMJD6 was amplified by using MCF-7 (ATCC, Manassas, VA, USA) mRNA by reverse transcriptase-polymerase chain reaction (RT-PCR) and was cloned into pCDH-CMV lentivirus vector containing a puromycin resistance gene (Transvector). Catalytic mutant JMJD6-mut (H187A) were generated with the QuikChange Lightning site-directed mutagenesis kit (Stratagene, La Jolla, CA, USA) according to the manufacturer’s instructions. All plasmid constructs described were verified by DNA sequencing. ADSCs were infected with the indicated viruses and selected with 1 μg/ml puromycin for 1 week.

### Differentiation

To induce osteogenic, chondrogenic and adipogenic differentiation of ADSCs, we cultured ADSCs JMJD6-Scr and ADSCs JMJD6-sh3 in three differentiation medias with StemPro Differentiation Kit (Gibco) according to the manufacturer’s instructions. Differentiation media were changed every 3 days. Oil-Red O (Sigma-Aldrich, St. Louis, MO, USA) staining was used to detect the lipid droplets. Toluidine Blue (Sigma-Aldrich) staining was processed for the evaluation of chondrogenic differentiation. The osteogenic differentiation was examined by staining for Alizarin Red (Sigma-Aldrich).

### Phenotype identification

Flow cytometry assay was performed to evaluate the expression of the MSCs markers. Stemflow hMSC Analysis Kit (BD Biosciences, Franklin Lakes, NJ, USA), was applied for flow cytometry assay, containing CD90 FITC (5E10), CD105 PerCP-Cy5.5 (266), CD73 APC (AD2), negative markers cocktail (CD45/CD34/CD11b/ CD19/HLA-DR-PE) and isotype controls. 1 × 10^6^ ADSCs cells were analyzed by using flow cytometer (FACSAria; BD Biosciences).

### siRNA transfection and migration screening

Transient siRNA transfections were performed using RNAiMAX (Invitrogen). Two days after the transfection, the ADSCs were used for migration assays. To profile histone demethylases genes associated with ADSCs migration, 5 × 10^4^ siRNA-transfected ADSCs in 0.5 ml suspension were added to upper chamber in triplicate. The chambers (membrane with 8 μm pore size) were placed in a 24-well plate and incubated at 37 °C, with 5% CO2 for 18 h. The underside of the chamber was fixed in methanol (Sigma-Aldrich) for 10 min and cells pre-stained with CFSE (Dojindo, Kumamoto, Japan), Three random fields were filmed by microscopy (Leica, Wetzlar, Germany) and cells were counted. The sequences of siRNAs are showed in Additional file 1: Table S1.

### RNA-seq assay

Total RNA was isolated by Trizol (Invitrogen) according to the manufacturer’s protocol. Three replicates of ADSCs control and ADSCs JMJD6 sh3 knockdown cells were used for the RNA-seq. A total of 5 μg RNA of each sample was used to prepare libraries. RNA sequencing was performed using HiSeq 4000 (Illumina, San Diego, CA, USA) by Novogene.

### RNA extraction, RT-PCR and qRT–PCR

The total cellular RNA was extracted from ADSCs performed using RNeasy mini kit (Qiagen, Venlo, Netherlands). RNA was incubated with DNase I (Invitrogen) to remove genomic DNA contamination. First-strand cDNA was synthesized from total RNA using SuperScript III first-strand synthesis system (Invitrogen). RT-PCR was performed with 10 ng of cDNA in 50 μl reaction volume containing gene-specific primers and Ex-Taq DNA polymerase (Takara, Tokyo, Japan). Quantitative reverse transcription-polymerase chain reactions (qRT-PCR) reactions were performed using PowerUp SYBR green master mix (Invitrogen) and 7300 real-time PCR system (ABI). The mRNA expression levels were normalized using β-actin RNA as internal control.

### Western blotting

Total cellular protein was extracted using the cell lysis buffer (Beyotime), and concentrations were determined by BCA protein assay kit (Beyotime). Lysates were loaded in SDS-AGE gel and electrophoresed. Proteins were transferred from gel to nitrocellulose membrane using a trans-blot electrophoretic transfer kit (Bio-Rad Laboratories, Hercules, CA, USA). Membranes were blocked in 5% skim milk in TBST buffer and incubated with primary antibodies anti-JMJD6 (1:3000; H-7, Santa Cruz Biotechnologies, Dallas, TX, USA), anti-histone H4R3me2a (1:3000; active motif), anti-HA tag (1:4000; HA.C5, Abcam), anti-α-tubulin (1:6000, DM1A, CST), anti-PDE1C (1:2000; Abcam) and anti-GAPDH (1:4000; 6C5, Abcam). After washing, the membranes were incubated with HRP goat anti-mouse IgG (Beyotime) or HRP goat anti-rabbit IgG (Beyotime). Membranes were then incubated in BeyoECL plus (Beyotime) and then imaged using Chemidoc imaging system (Bio-Rad Laboratories). Gray value was analyzed using Image J (Java 1.8.0).

### Wound healing assay

Six-well cell culture plates were coated with 10 μg/cm^2^ collagen I (Sigma-Aldrich), 1 × 10^6^ cells were cultured in pre-coated wells at 37 °C with 5% CO_2_ for 24 h. A 200 μl yellow pipette tip was used to scratch the confluent cells monolayer. The mediums were changed with fresh medium and the scratches were analyzed after 20 h using ImageJ software.

### Cell proliferation assay

5000 cells in 100 μl α-MEM with 10% FBS were plated in 96-well cell culture plates. At different time points, 10 μl CCK8 regent (Dojindo) was added to the cell culture medium and incubated at 37 °C with 5% CO_2_ for 1 h. The absorbance of cell suspension was measured at 450 nm using a microplate reader (Bio-Rad Laboratories). Cells number was determined according to the standard curve. For EdU assay, the cells were plated on poly-lysine-coated coverslips in a 24-well cell culture plates and incubated at 37 °C with 5% CO_2_ overnight. 10 μM EdU solution (Invitrogen) was added to the cell culture medium treated for 6 h. Then coverslips were fixed using 3.7% formaldehyde in PBS and permeabilization by a 0.5% Triton X-100 solution. 0.5 ml of Click-it plus reaction cocktail (Invitrogen) was added to each coverslip and incubated for 30 mins. Hoechst 33,342 (Invitrogen) was used to identify nucleus. Coverslips were treated with mounting media and imaged by fluorescence microscopy (Leica).

### Cell cycle assay

Cells were resuspended with trypsin, washed in PBS and fixed in cold 70% ethanol at 4 °C for 30 min. 50 μl of 100 μg/ml RNase was added to eliminate RNA. Then cells were incubated with 425 μl cell staining buffer (Biolegend) and 25 μl propidium iodide solution (Biolegend). Cell DNA contents were analyzed using flow cytometry (FC500, Beckman Coulter, Brea, CA, USA).

### ChIP qPCR

Chromosome immunoprecipitation (ChIP) assays were performed using SimpleChIP plus enzymatic chromatin IP kit (CST). 4 ×10^6^ cells were fixed with 540 μl of 37% formaldehyde to cross-link proteins with DNA. Chromatin was digested with 0.5 μl micrococcal nuclease into 150-900 bp DNA/protein fragments. Then 10 μl antibody H4R3me2a (active motif) was added to the IP sample and the complex co-precipitates was captured by protein G agarose. Cross-links were reversed, and DNA was purified using spin column and proceeded for qPCR analysis. The primers used for amplifying the promoter are provided in Additional file 1: Table S1.

### cAMP and cGMP determination assay

Cyclic hydrolysis both cyclic adenosine monophosphate (cAMP) and cyclic guanosine monophosphate (cGMP) levels in ADSCs were measured using cyclic AMP and GMP XP Assay kit (CST, Danvers, MA, USA). 7 × 10^3^ ADSCs were cultured with α-MEM and treated with 50 μM vinpocetine (Sigma-Aldrich). After 24 h, the cells were rinsed twice with PBS, and lysed with lysis buffer. 50 μl cell lysate with 50 μl of the HRP cAMP solution were added to the cAMP assay plate. Add 100 μl TMB substrate, incubate for 30 min, and absorbance were measured at 450 nm using a microplate reader (Bio-Rad Laboratories).

### Statistical analysis

We used the GraphPad Prism software (v7) to perform statistical analysis (GraphPad Software, San Diego, CA, USA). Data were expressed as the mean ± SD. Unless otherwise indicated, differences between two experimental groups were applied using an unpaired two-tailed Student’s *t* test. For comparison more than three groups, one-way ANOVA was applied. Results were considered statistically significant with *p* values: **p < 0.01; *p < 0.05.

## Results

### JMJD6 is critical for cell migration

**Fig. 1:**
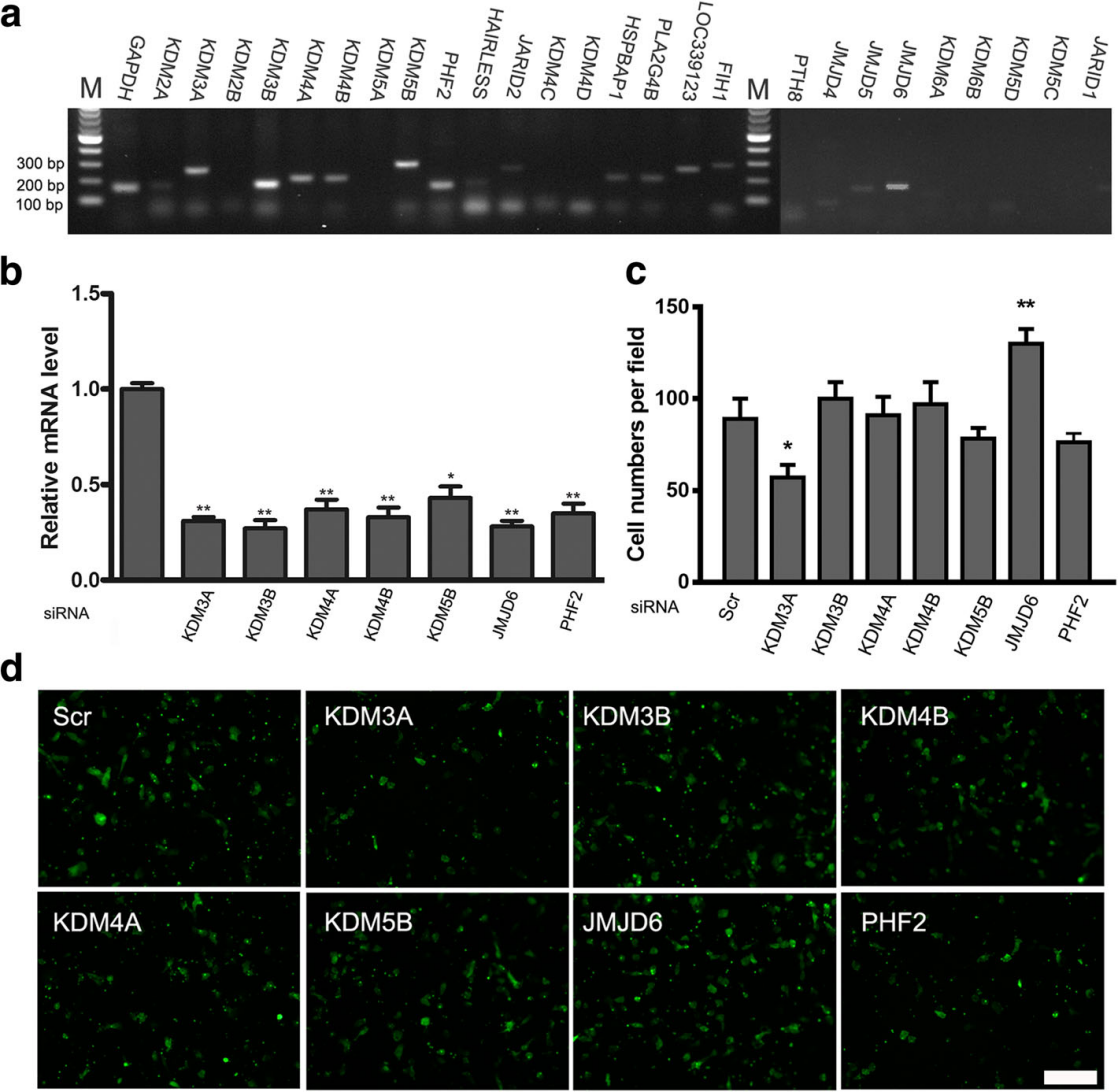
JMJD6 knockdown elevates migration in ADSCs. **a** Histone demethylase gene expression profiling in ADSCs. The mRNA expression of 27 histone demethylases was measured using RT-PCR. **b** qRT-PCR analysis of histone demethylase gene expression. ADSCs were transfected with targeted siRNAs, fold changes are relative to that of ADSCs treated with the scrambled (Scr) siRNA. **c** and **d** Cell migration assay showing the relationship between profiled histone demethylases and cell migration. ADSCs were treated with different siRNAs and were allowed to migrate to α-MEM with 20% FBS. After 18 h, CFSE prestained cells were fixed and counted. Scale bar = 200 μm, magnification = ×100, *n* = 3/group, Error bars represent the SD. **p < 0.01; *p < 0.05 versus the controls. *ADSCs* adipose-derived mesenchymal stem cells, *JMJD6* Jumonji C domain-containing protein 6

To investigate the potential roles of histone demethylases in the regulation of ADSCs migration, we first profiled the expression of 27 histone demethylases in ADSCs. RT-PCR assays showed that the ADSCs used in our experiments express histone demethylases, including KDM3A, KDM3B, KDM4A, KDM4B, KDM5B, JMJD6, and PHF2 (Fig. 1a). Next, we transfected ADSCs with small interfering RNA (siRNA) against these histone demethylases, or with control siRNA, and confirmed the knockdown efficacy only by qRT-PCR, western blot assay was not employed. The results revealed independent histone demethylase targeted siRNAs reduced the mRNA levels significantly compared to control (Fig. 1b). We also examined the ADSCs migration among the differentially treated groups, and found that it increased significantly (by about 130 ± 8 cells/field with) when JMJD6 was knocked down, while KDM3A depletion inhibited migration (Fig. 1c and d), demonstrating that JMJD6 knockdown promoted the migration of ADSCs. Thus, JMJD6 emerged as the top candidate associated with the regulation of ADSCs migration and was selected for further investigation.

**Fig. 2:**
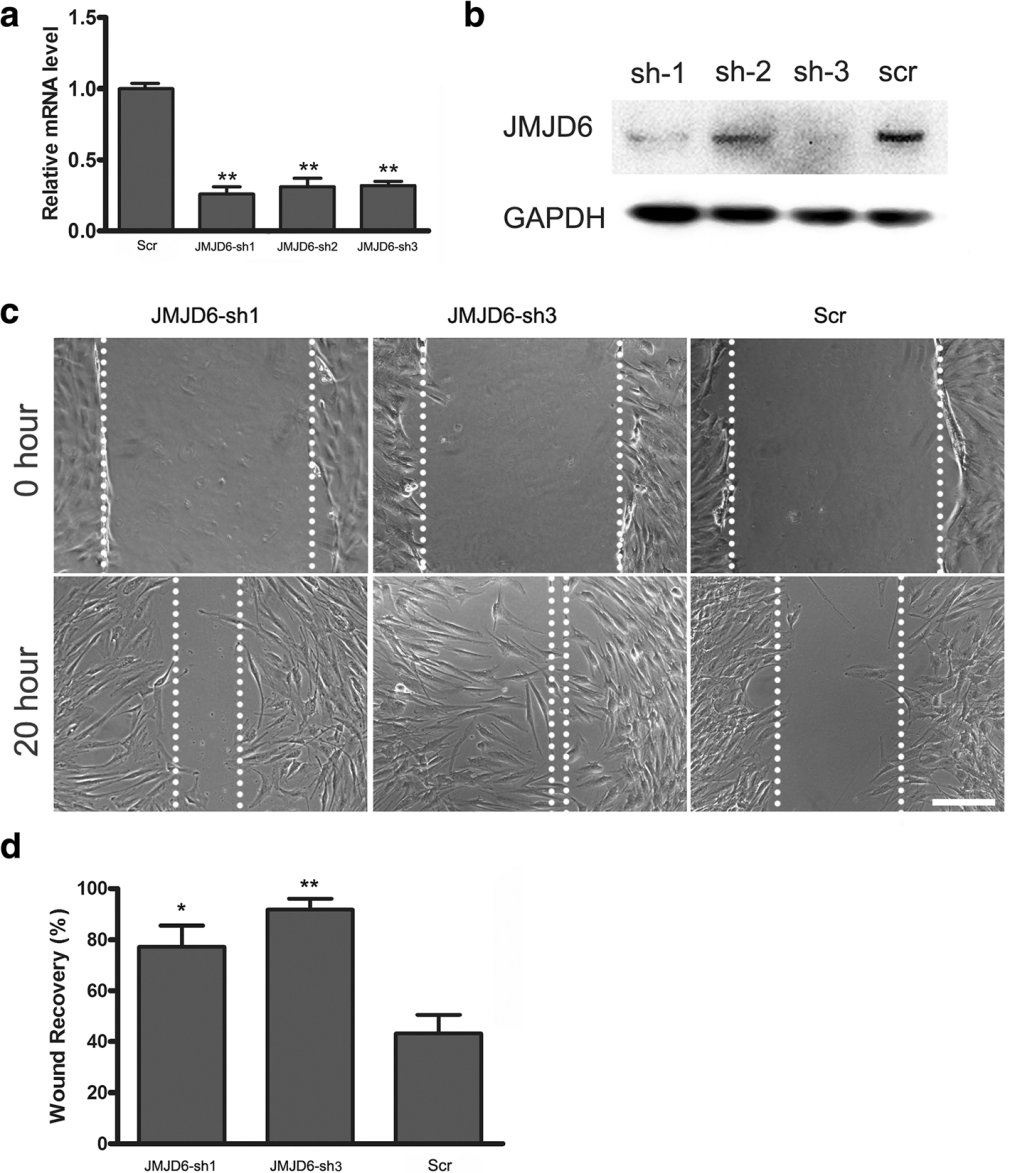
JMJD6 depletion promotes the wound-healing ability of ADSCs. **a** The shRNA mediated knockdown of JMJD6 in ADSCs. The JMJD6 mRNA expression level was determined by qRT-PCR. **b** ADSCs were infected with the viruses and selected with puromycin for 1 week. Next, the JMJD6 protein expression levels were examined by western blot experiments. **c** and **d** Wound-healing assay for JMJD6 knockdown ADSCs compared with ADSCs transfected with the scrambled (Scr) sequence. The graph presents the ratios of wound recovery of JMJD6 knockdown ADSCs relative to the control group. n = 3/group, Error bars represent the SD. **p < 0.01; *p < 0.05versus the control. Scale bar = 200 μm, magnification = ×100. *ADSCs*: adipose-derived mesenchymal stem cells, *JMJD6* Jumonji C domain-containing protein 6

To confirm whether JMJD6 depletion promotes ADSCs migration and to determine the underlying mechanism of JMJD6-mediated epigenetic regulation, we utilized three lentivirus-based short hairpin RNAs (shRNA) to specifically target different sequences on the JMJD6 mRNA. As shown in (Fig. 2a and b), qRT-PCR and western blot experiments demonstrated that the JMJD6 mRNA and protein levels were reduced by more than 70% in ADSCs expressing JMJD6 sh1 and JMJD6 sh3 as compared to ADSCs expressing the scrambled shRNA (Fig. 2b). Consistent with our results from the siRNA-mediated JMJD6 knockdown, ADSCs with JMJD6 depletion resulted in a striking increase in wound-healing ability as compared to cells transfected with the control shRNAs (Fig. 2c).

### JMJD6 regulates ADSCs proliferation

**Fig. 3:**
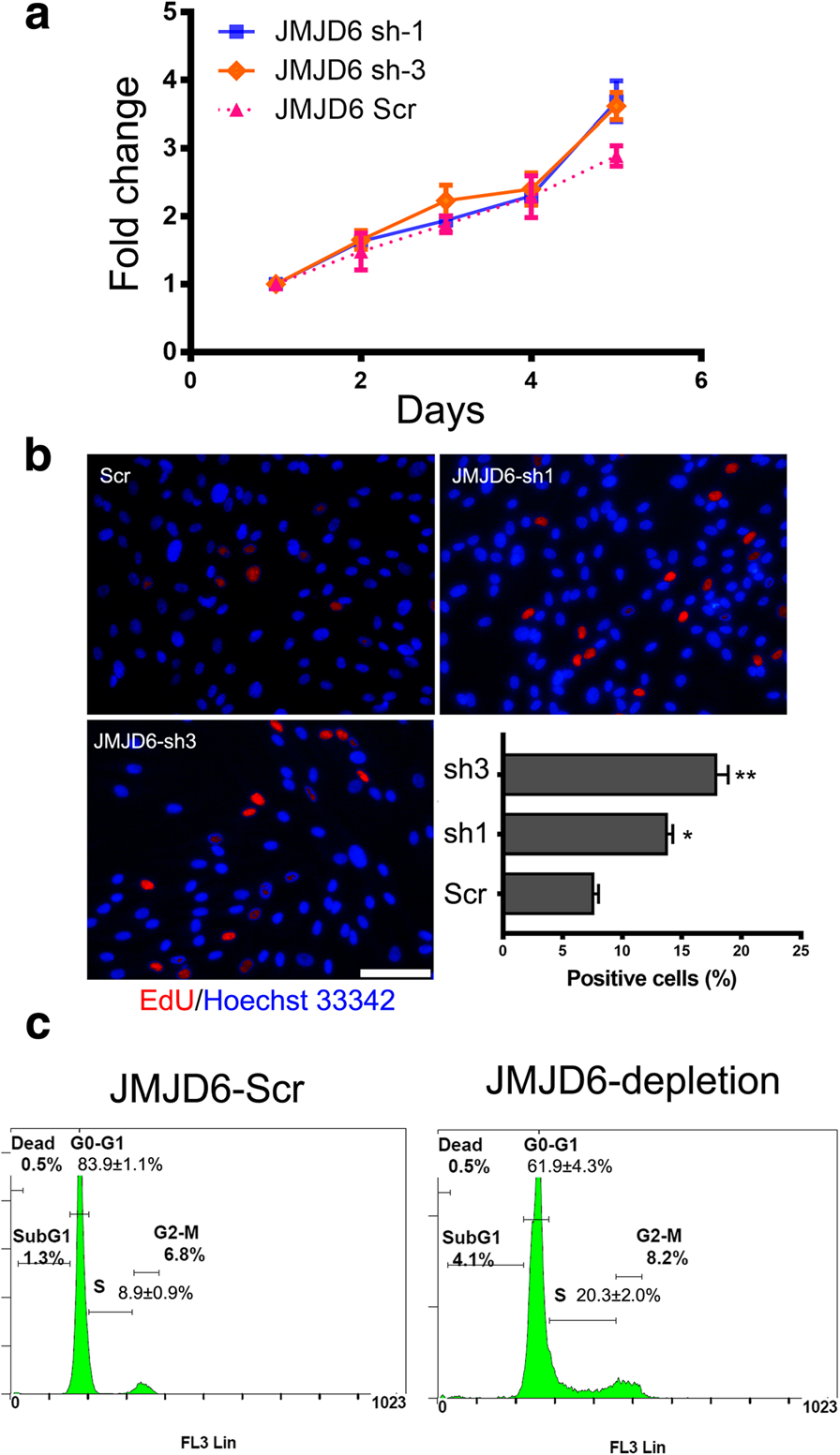
JMJD6 depletion facilitates ADSCs proliferation. **a** Cell proliferation assay. The CCK8 assay was used to determine ADSCs cell number based on the standard curve. The absorbance of the cell suspension was measured at 450 nm. **b** Cell proliferation was assessed with an EdU labeling assay in JMJD6 depletion ADSCs and control cells. The images display EdU staining (*red color*) merged with Hoechst 33,342 staining (*blue color*). The graph represents as the percentage of EdU-positive cells. **c**. Cell cycle analysis. JMJD6 depleted ADSCs and control cells were incubated with propidium iodide staining solution and the cellular DNA contents were analyzed by flow cytometry. *n* = 3/group, Scale bar = 200 μm, magnification = ×100, Error bars represent the SD, **p < 0.01; *p < 0.05 versus the control group by the Student’s *t* test. *ADSCs* adipose-derived mesenchymal stem cells, *EdU* 5-ethynyl-2′-deoxyuridine, *JMJD6* Jumonji C domain-containing protein 6, *Sh1* ADSCs-JMJD6 sh1, *Sh3* ADSCs-JMJD6 sh3, *Scr* ADSCs-JMJD6 scr

To determine the role of JMJD6 in ADSCs proliferation, the JMJD6 sh1 and sh3 stably transduced ADSCs cell lines were employed. The proliferation rate was measured with a CCK8 assay. JMJD6 depletion caused a significant increase in the ADSCs proliferation rate as compared to the control cells (Fig. 3a). Similarly, the BrdU labeling assay, which is frequently used as a reliable way to label actively dividing cells, showed that the percentage of BrdU-positive cells was markedly increased in ADSCs-JMJD6 sh3 and ADSCs-JMJD6 sh1 cells as compared to the control ADSCs-JMJD6 scr cells (Fig. 3b). Since the depletion of JMJD6 profoundly increased the proliferation rate of ADSCs, we extended our analysis to explore the cell cycle status of ADSCs by assessing their DNA content using PI staining analysis. As shown in (Fig. 3c), the flow cytometry experiments demonstrate that JMJD6 depletion in ADSCs results in a remarkable decrease of cells in the G0/G1 phase (from 83.9 ± 1.1% to 61.9 ± 4.3%), indicating fewer cells in the quiescent state. The results also revealed that JMJD6 knockdown promotes cell cycle progression into the S phase (20.3 ± 2.0% cells vs. 8.9 ± 0.9% cells in the control group). However, ADSCs differentiation assays and flow cytometry analysis revealed that the JMJD6 knockdown in ADSCs did not affect their differentiation capacity and MSCs cell surface marker expression (Additional file 2: Figure S1 and Additional file 3: Figure S2). Taken these results together, these data suggest that the depletion of JMJD6 in ADSCs can contribute to cell proliferation in vitro.

### RNA-seq analysis of JMJD6 knockdown ADSCs

**Fig. 4:**
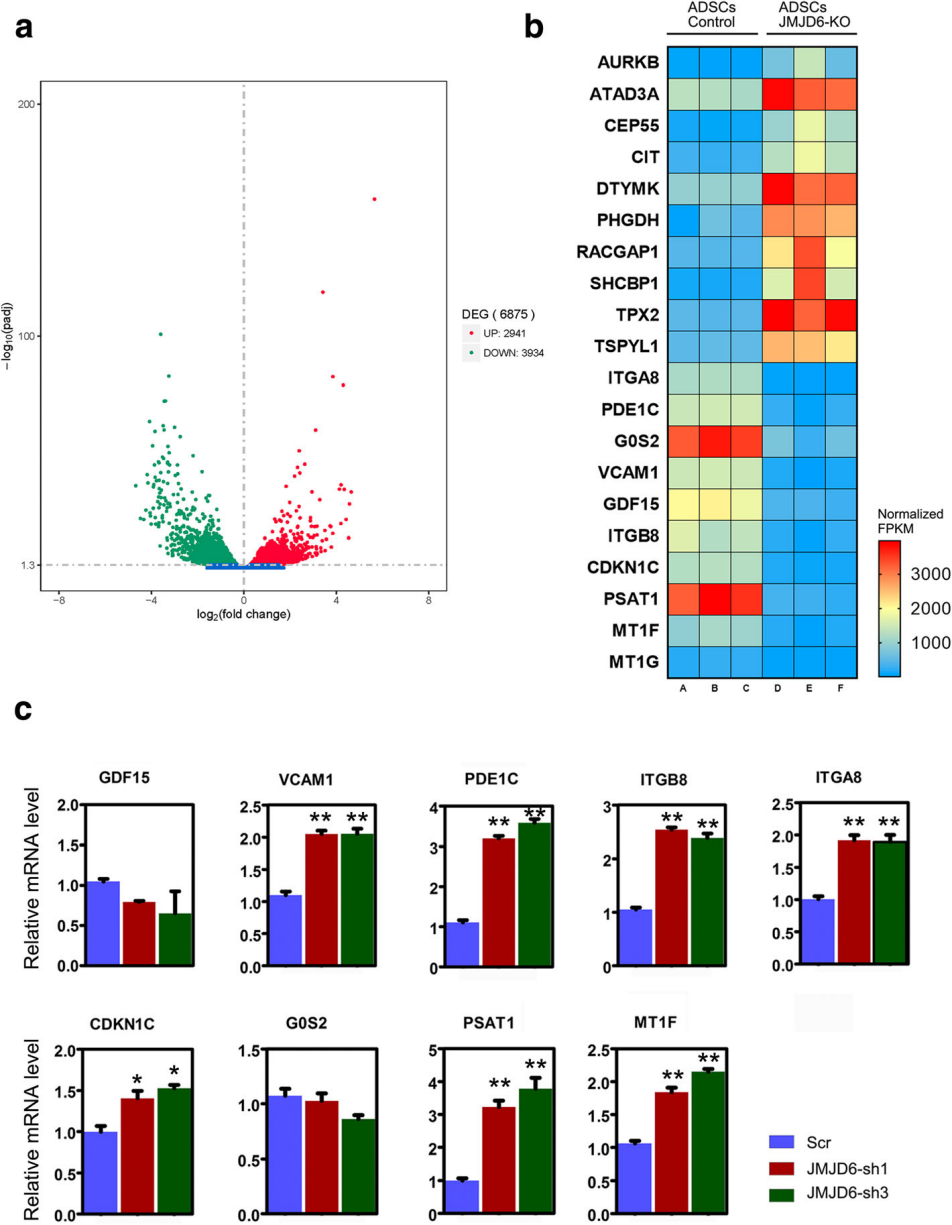
Gene expression changes in JMJD6-knockdown ADSCs. **a** Volcano plot analysis of differential gene expression (log2-fold changes and the corresponding *p* values for each gene). *Red dots* represent upregulated genes (*n* = 3934) and *green dots* represent downregulated genes (*n* = 2941). **b** The heatmap shows the FPKM value changes of the top 10 genes (up and downregulated) related to the cell cycle and cellular movement from control ADSCs and JMJD6-depleted ADSCs. Each *colored square* represents a biological replicate, *n* = 3. **c** qRT-PCR analysis was performed to validate the expression changes of the indicated genes in JMJD6 knockdown ADSCs. The y-axis represents the relative mRNA fold change, which is calculated using the 2^−ΔΔCt^ formula with β-actin as internal control. Error bars represent the SD. *ADSCs* adipose-derived mesenchymal stem cells, *FPKM* fragments per kilobase per million, *JMJD6* Jumonji C domain-containing protein 6

It is known that JMJD6 is a known epigenetic regulator, capable of removing methyl groups from histone dimethyl symmetric H4R3 (H4R3me2s), which is associated with gene silencing and can also remove methyl groups from histone dimethyl asymmetric H4R3 (H4R3me2a), which is often associated with active gene transcription [20, 25]. More specifically, JMJD6 is involved in numerous functions, including angiogenic sprouting [26], cellular transformation [27], and cellular proliferation and motility [28]. The underlying molecular mechanisms of this arginine demethylase, JMJD6, remain debatable and its role in ADSCs remains to be fully elucidated. To identify the changes in gene expression between virally transduced JMJD6-depleted and control ADSCs, RNA-seq analysis was carried out. The results demonstrate that 2941 genes were upregulated and 3934 genes were downregulated following JMJD6 depletion in ADSCs (Fig. 4a). To further explore the molecular and cellular functions associated with JMJD6 depletion, Ingenuity Pathway Analysis (IPA) was performed. The top enriched functions were cell cycle and cellular movement, followed by cellular assembly and organization (Table 1). A heat map illustrates the fold change in the expression levels of 20 genes corresponding to the top enriched function determined by RNA-seq (Fig. 4b). Among the associated gene functions, we selected ITGA8, ITGB8, G0S2, CDKN1C, PSAT1, PDE1C, GDF15, VCAM1, and MT1, all shown to be related to cellular proliferation and migration, which was further confirmed by qRT-PCR experiments. Most differentially expressed genes in the JMJD6-depleted ADSCs were upregulated as compared to the control cells (Fig. 4c).

**Table 1:** Molecular and cellular functions

Name	*p* value	#Genes
Cell cycle and cellular movement	2.17E-05 - 9.62E-44	196
Cellular assembly and organization	8.80E-06 - 9.42E-34	172
DNA replication and repair	1.70E-05 - 9.42E-34	161
Cell death and survival	2.30E-05 - 3.82E-21	233
Cell morphology	7.01E-06 - 6.68E-20	105

IPA analysis identified top five genes enrichments of the molecular and cellular functions in JMJD6-knockdown ADSCs compared with ADSCs with control shRNA

### JMJD6 promotes PDE1C expression in a demethylase dependent manner

Next, we set out to determine how JMJD6 depletion facilitates the transactivation of PDE1C, which was significantly increased gene in ADSCs cells after JMJD6 depletion. PDE1C is typically regarded as a regulator of cyclic hydrolysis for cyclic adenosine monophosphate (cAMP) and cyclic guanosine monophosphate (cGMP). According to the latest knowledge, PDE1C can modulate the migration [29], and proliferation of arterial smooth muscle cells (ASMCs) [30], as well as endometriosis [31]. Furthermore, PDE1C plays a critical role in the regulation of glioblastoma growth and migration [32]. Thus, we further explored whether PDE1C was targeted by JMJD6 in the regulation of ADSCs migration and proliferation.

**Fig. 5:**
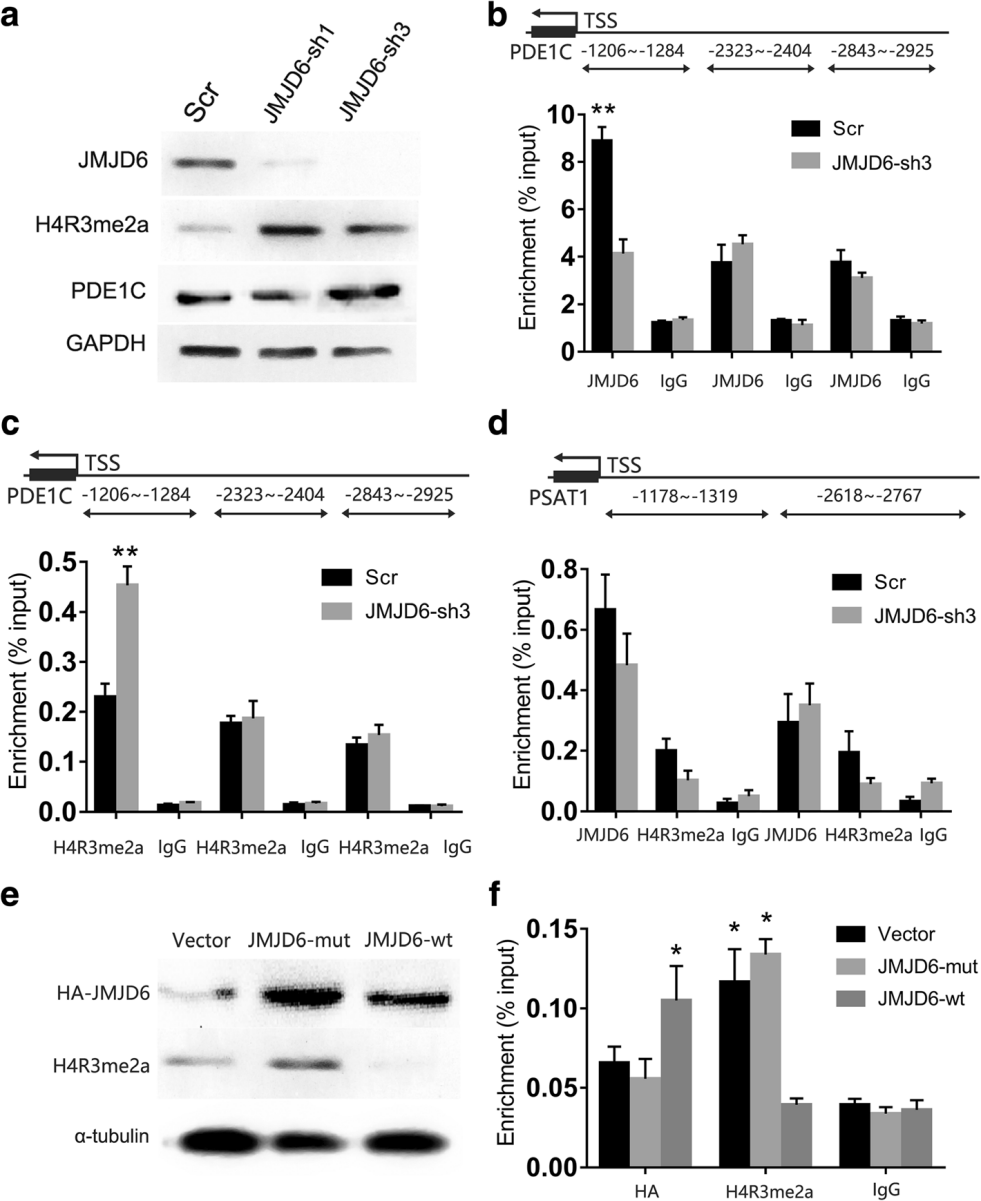
JMJD6 regulates the expression of PDE1C by demethylating H4R3me2a at its promoter. **a** Western blot analysis of JMJD6, histone modifications, and PDE1C in JMJD6 knockdown ADSCs. **b** ChIP assays of JMJD6 and (**c**) H4R3me2a binding at the PDE1C promoter in JMJD6-depleted ADSCs or Scr control ADSCs. Primers used for the ChIP assays are shown as a schematic map at the top of the figures. **d** ChIP analysis of JMJD6 and H4R3me2a occupancies on regions near the PDE1C promoter in JMJD6- depleted and Scr control ADSCs. **e** Western blot analysis of JMJD6 and histone modifications in JMJD6 rescued cell lines. **f** ChIP assays of JMJD6 and H4R3me2a binding at the PDE1C promoter in JMJD6 rescue cell lines. Error bars represent the SD. *TSS* transcriptional start site. *ADSCs* adipose-derived mesenchymal stem cells, *AMP* cyclic hydrolysis both cyclic adenosine monophosphate, *cGMP* cyclic guanosine monophosphate, *H4R3me2a* histone dimethyl asymmetric H4R3, *PDE1C* phosphodiesterase 1C, *JMJD6* Jumonji C domain-containing protein 6

Western blot experiments showed that JMJD6 depletion led to a global increase in H4R3me2a levels, suggesting that JMJD6 is the main H4R3me2a demethylase in ADSCs. The results also revealed that PDE1C expression is regulated by JMJD6 in ADSCs (Fig. 5a).

The gray value of PDEC1 in JMJD6-sh1 (11792) and JMJD6-sh3 (42810) was higher than that of the cells transfected with the scrambled vector (9358). Next, we set out to how JMJD6 epigenetically modulate PDE1C expression. ChIP assays indicated that JMJD6 binds upstream of PDE1C promoter (Fig. 5b). As expected, the H4R3me2 antibody targeted the same region of the PDE1C promoter and showed a 2.2-fold higher enrichment in JMJD6-sh3 cells as compared to the JMJD6-Scr cells (Fig. 5c). However, the results did not indicate JMJD6 binding at the PSAT1 promoter (Fig. 5d). To validate these observations, we generated three JMJD6 rescue cell lines, including ADSCs-vector, ADSCs-JMJD6-mut (H187A), and ADSCs-JMJD6-wt cells. Western blot experiments demonstrated that the protein expression levels of wild-type JMJD6 were similar to the expression of its catalytic mutant JMJD6-mut (H187A). However, only the expression of wild-type JMJD6 in ADSCs-JMJD6-wt cells, but not of its catalytic mutant, JMJD6-mut (H187A), significantly decreased H4R3me2a expression (Fig. 5e), indicating that the catalytic mutant, JMJD6-mut (H187A) is unable to induce H4R3me2a demethylation. In addition, H4R3me2a enrichment decreased in ADSCs-JMJD6-wt cells, but remained in ADSCs-vector and ADSCs-JMJD6-mut cells. Consistent with our ChIP assay results, JMJD6 rescue suppressed the enrichment of H4R3me2a at the PDE1C promoter (Fig. 5f), suggesting that JMJD6 suppresses PDE1C expression through the inducing H4R3me2a demethylation at the PDE1C promoter.

### JMJD6 regulates ADSCs migration and proliferation through PDE1C

**Fig. 6:**
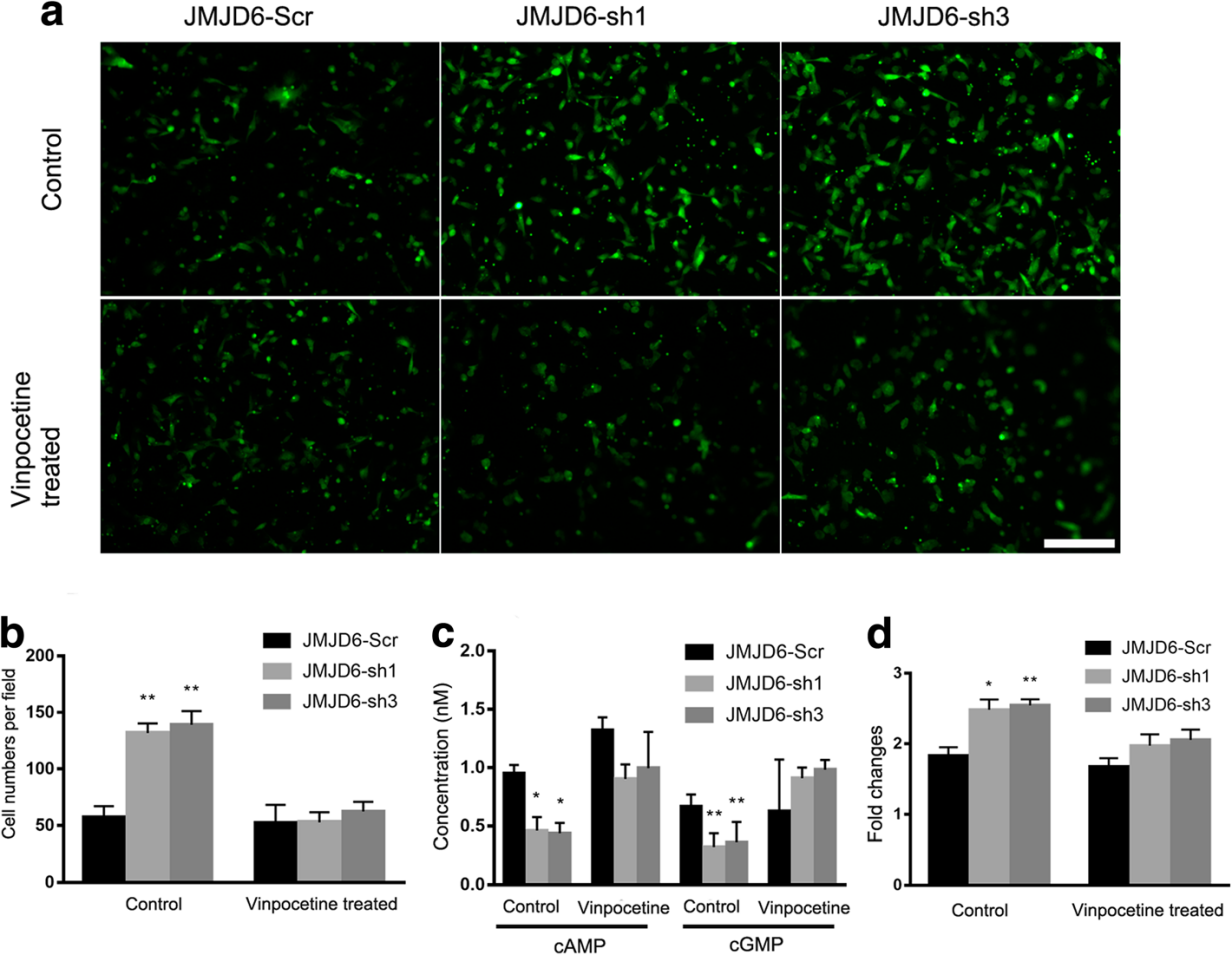
The PDE1C inhibitor vinpocetine depresses cellular migration and proliferation in JMJD6-knockdown ADSCs. **a** Transwell migration assays show ADSCs prestained with CFSE after vinpocetine treatment. The migration capability of JMJD6-knockdown but not the JMJD6-Scr ADSCs is dramatically reduced. **b** The graph represents the number of migrated cells. **c** Intracellular levels of cAMP and cGMP increased significantly in the JMJD6-knockdown ADSCs but not in JMJD6-Scr ADSCs after treatment with the PDE1C inhibitor vinpocetine. Concentrations are expressed the means ± SD of three independent experiments. **d** Cell proliferation was determined according to the standard curve using a CCK8 assay; vinpocetine treatment caused a significant decrease in the proliferation rate of JMJD6-knockdown ADSCs as compared to the JMJD6-Scr ADSCs. Scale bar = 200 μm, n = 3/group, magnification = ×100, Error bars represent SD, **p < 0.01;*p < 0.05 versus the vinpocetine-treated group. *ADSCs* adipose-derived mesenchymal stem cells, *cAMP* cyclic hydrolysis both cyclic adenosine monophosphate, *cGMP* cyclic guanosine monophosphate, *H4R3me2a* histone dimethyl asymmetric H4R3, *PDE1C* phosphodiesterase 1C, *JMJD6* Jumonji C domain-containing protein 6

To determine whether the alteration of PDE1C expression due to JMJD6 depletion effects the migration and proliferation of ADSCs, we employed the PDE1C inhibitor vinpocetine. Consistent with our transwell migration assay results, JMJD6 depletion resulted in increased ADSCs migration (Fig. 6a and b). Following vinpocetine treatment, the cellular proliferation and motility were significantly attenuated in the JMJD6-depleted ADSCs (Fig. 6c and d), suggesting that this regulation could be partially attributed to the increase of PDE1C expression levels in the JMJD6-depleted ADSCs. PDE1C is a phosphodiesterase that hydrolyzes cAMP and cGMP to 5′AMP and 5′GMP. The secondary messengers, cAMP and cGMP are known to be involved in the regulation of cellular proliferation and differentiation in various cell types, including embryonic stem cells (ESs) [33], dendritic cells (DCs) [34], and epithelial cells [35]. To further understand the mechanisms by which the upregulation of PDE1C led to increased cellular proliferation and migration, we analyzed the levels of cAMP and cGMP in control and JMJD6-depleted ADSCs. The results demonstrate that the cAMP and cGMP level in control ADSCs were 0.948 ± 0.06 nM and 0.666 ± 0.08 nM respectively. In JMJD6-depleted ADSCs, cAMP and cGMP levels were dramatically dropped to (0.461 ± 0.09 nM and 0.322 ± 0.09 nM) in JMJD6-sh1 cells and (0.437 ± 0.07 nM and 0.364 ± 0.13 nM) in JMJD6-sh3 cells. In JMJD6-depleted ADSCs treated with the PDE1C inhibitor, the cAMP and cGMP levels were increased to (0902 ± 0.1 nM and 0.908 ± 0.08 nM) in JMJD6-sh1 cells and (0.995 ± 0.25 nM and 0.982 ± 0.07 nM) in JMJD6-sh3 cells (Fig. 6c), consistent with the decreased proliferation and motility observed. In total, these data suggest that cAMP and cGMP act as the downstream components of the JMJD6-mediated epigenetic regulation of PDE1C.

## Discussion

Recently, studies focusing on ADSCs have gradually shifted from animal models into clinical trials, indicating that ADSCs are a promising therapeutic tool [36–38]. It is known that ADSCs proliferation and motility are critical for the benefit of stem cells based cytotherapy [39, 40]. However, the regulation of these critical functions remains poorly understood, and our primary goal in this study was to better characterize how they are regulated. Various molecules and signaling pathways are involved in the regulation of ADSCs migration and proliferation, including MCP-1, SDF-1, Wnt, and Jun amino-terminal kinase (JNK) [41, 42]. These regulatory processes are complex and involve multiple genes and mechanisms. Recent studies have shown that epigenetic regulation is important in modulating MSCs self-renewal and differentiation [43, 44]. In the present study, we identified JMJD6 as an epigenetic regulator involved in controlling the proliferation and migration of ADSCs by removing methyl groups from H4R3me2a at the PDE1C promoter.

JMJD6, also termed phosphatidylserine receptor (PTDSR), has been shown to function as a cell-surface protein that engages and mobilizes phagocytic cells [45]. Recently, it was determined that JMJD6 serves two distinct functions as a histone arginine demethylase and a lysyl oxidase [20, 46]. Through a histone demethylase knockdown profile assay, we confirmed JMJD6 as an epigenetic regulator associated with cell motility. Furthermore, using RNA-seq followed by pathway analysis, we discovered that JMJD6-depleted ADSCs exhibited enrichment gene programs associated with cell cycle and cell movement. This was consistent with our findings that, shRNA-mediated depletion of JMJD6 in ADSCs promoted cell cycle progression into the S phase and increased the number of proliferating cells. Our ChIP-qPCR results indicated that the depletion of JMJD6 promotes PDE1C expression and cell migration via a histone demethylase dependent mechanism. Restoration of wild-type JMJD6 expression, but not its catalytic mutant JMJD6-mut (H187A) attenuated the H4R3me2a level and the enrichment of H4R3me2a on the PDE1C promoter in ADSCs, indicating that JMJD6 demethylase activity is directly related to PDE1C expression. Although there was a strong enrichment of JMJD6 at the PDE1C promoter, we did not observe JMJD6 at the PSAT1 promoter, which has been shown to promote cell cycle progression and cell proliferation by inhibition of cyclin D1 degradation [47].

As a cyclic nucleotide phosphodiesterases, PDE1C is expressed in limited cell types, including epithelial cells [48], pancreatic beta-cells [49], vascular smooth muscle cells (VSMCs) [50] and smooth muscle cells (SMCs) [29]. Interestingly, previous studies have shown that PDE1C is a proliferation-driving gene that is only expressed in dividing VSMCs and SMCs. In addition, PDE1C can hydrolyze both cAMP and cGMP [49], which are intracellular secondary messengers, whose related functions are regulated by PDE1C. More importantly, cAMP and cGMP have important roles in many processes from cellular proliferation to stem cell differentiation [51, 52]. Cellular maintenance of an appropriate level of cAMP and cGMP is critical. For example, increasing the intracellular cAMP levels can lead to cardiomyocyte differentiation and inhibition of proliferation in embryonic stem cells [52]. Our data demonstrate that the knockdown of JMJD6 in ADSCs can induce a decrease in cAMP and cGMP levels. However, the differentiation ability and MSCs cell surface marker expression was not significantly affected. Currently, little is known about the role of PDE1C in ADSCs and about the specific mechanisms of how PDE1C can regulate their proliferation and migration. In this study, using shRNA-mediated JMJD6 depletion in ADSCs and chemical inhibition of PDE1C by vinpocetine, we demonstrated that the increase in PDE1C expression, induced by loss of JMJD6, facilitates the proliferation and migration of ADSCs, and is associated with lower levels of both cAMP and cGMP. All these effects can be suppressed by inhibition of PDE1C with vinpocetine including the rescue of cAMP and cGMP levels, suggesting that PDE1C’s effect on the proliferation and migration of ADSCs is dependent cAMP and cGMP activity. The mechanisms that govern the migration of transplanted cells remain unclear. If the JMJD6-PDE1C axis contributes to this process, the epigenetic regulation may accelerate ADSCs migration to the injury site and facilitate tissue repair. Our findings offer a new perspective on the role of JMJD6 as a key epigenetic regulator in ADSCs, which can be relevant for cell-based therapies.

## Conclusions

**Fig. 7:**
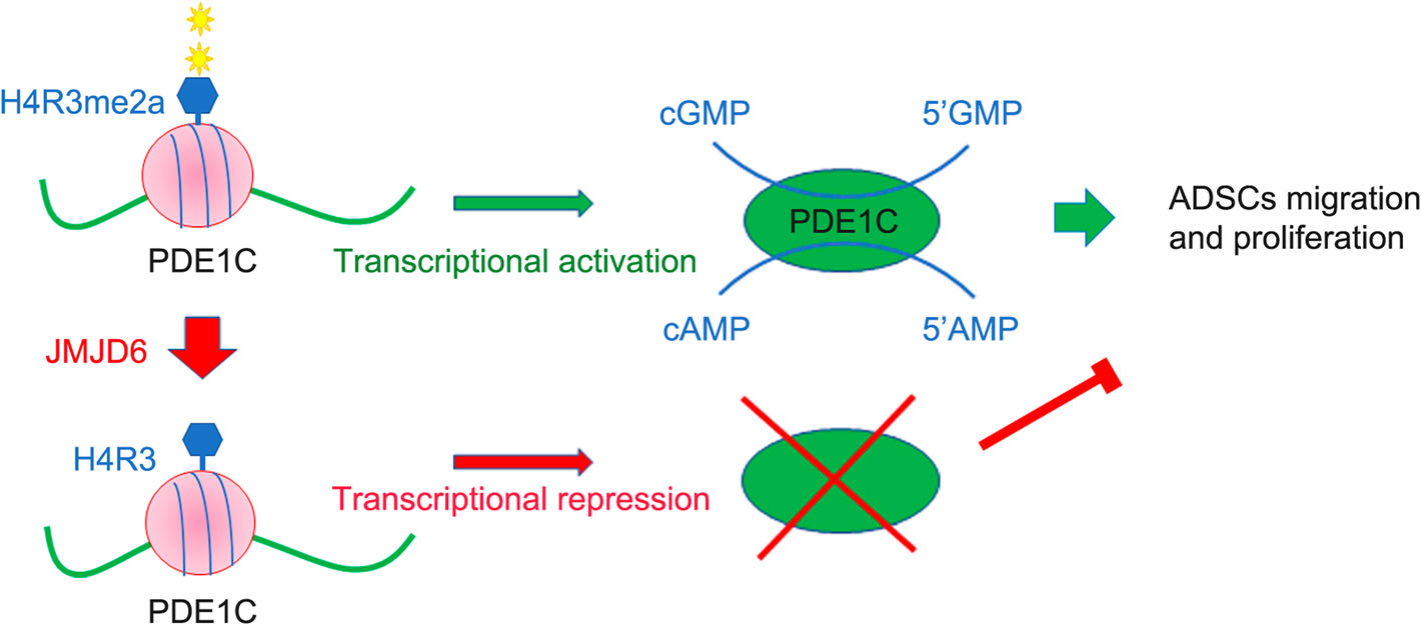
JMJD6 as an epigenetic regulator modulate PDE1C-cAMP/cGMP-dependent proliferation and migration in ADSCs. JMJD6 is the main H4R3me2a demethylase in ADSCs that epigenetically modulate PDE1C expression by binding upstream of PDE1C promoter and demethylating H4R3me2a at the PDE1C promoter. PDE1C can hydrolyze both cAMP and cGMP into 5’ AMP and 5’ GMP, which are intracellular secondary messengers, whose related functions are regulated by PDE1C. *ADSCs* adipose-derived mesenchymal stem cells, *cAMP* cyclic hydrolysis both cyclic adenosine monophosphate, *cGMP* cyclic guanosine monophosphate, *H4R3me2a* histone dimethyl asymmetric H4R3, *PDE1C* phosphodiesterase 1C, *JMJD6* Jumonji C domain-containing protein 6

In summary, our data indicate that JMJD6 regulates the proliferation and migration of ADSCs by demethylating H4R3me2a at PDEC1 promoter regions and consequently suppressing PDE1C expression. Inhibition of PDE1C in ADSCs leads to attenuation of cellular proliferation and motility. We also, validated that the cAMP and cGMP are important signaling molecules downstream of PDE1C (Fig. 7). Our work identifies JMJD6 as a key epigenetic regulator of ADSCs function. The regulatory interplay between JMJD6, PDE1C and the cAMP/cGMP pathway provides possible opportunities to modulate the characteristics of ADSCs to facilitate their clinical applications.

## Additional files

Additional file 1: **Table S1.** The sequences of shRNAs targeting JMJD6, siRNAs targeting KDM3A, KDM3B, KDM4A, KDM4B, KDM5B, JMJD6 and PHF2. The qPCR primers and the primers used for ChiP. (DOCX 18 kb)

Additional file 2: **Figure S1.** Effects of JMJD6 loss on cell surface marker expression in ADSCs. The graphs show the ADSCs surface antigen phenotypes. Positive expression markers: CD90-FITC, CD73-APC, and CD105-PerCPCy5.5. Negative expression markers: (CD45/CD34/CD11b/CD19/ HLA-DR PE). (TIF 407 kb)

Additional file 3: **Figure S2.** The osteogenic, adipogenic, and chondrogenic differentiation of ADSCs. After ADSCs were cultured in vitro for 16 days, osteogenic differentiation was analyzed by Alizarin Red staining (a, b). Adipogenic differentiation was measured by Oil Red O staining (c, d) and chondrogenic differentiation capacity was examined by Toluidine Blue staining (e, f). Scale bar = 200 μm, magnification = ×50. (TIF 9808 kb)

## Abbreviations

ADSCs: Adipose-derived mesenchymal stem cells
BM-MSCs: Bone marrow mesenchymal stem cells
cAMP: Cyclic hydrolysis both cyclic adenosine monophosphate
cGMP: Cyclic guanosine monophosphate
H4R3me2a: Histone dimethyl asymmetric H4R3
H4R3me2s: Histone dimethyl symmetric H4R3
JMJD6: Jumonji C domain-containing protein 6
MSCs: Mesenchymal stem cells
PDE1C: Phosphodiesterase 1C
shRNA: Short hairpin RNA
siRNA: Small interfering RNA
