# Supplementary material for: Histone demethylase JMJD6 regulates cellular migration and proliferation in adipose-derived mesenchymal stem cells
Source: Stem Cell Res Ther. 2018 Aug 9;9:212. doi: 10.1186/s13287-018-0949-3 (PMC6085710; doi:10.1186/s13287-018-0949-3)
Supplement: Supplementary file 1 — Table S1. The sequences of shRNAs targeting JMJD6, siRNAs targeting KDM3A, KDM3B, KDM4A, KDM4B, KDM5B, JMJD6 and PHF2. The qPCR primers and the primers used for ChiP. (DOCX 18 kb) [file 13287_2018_949_MOESM1_ESM.docx]

**The** **sequences of shRNAs targeting JMJD6**

| Name | sequence |
| --- | --- |
| Sh1 | 5’-GGAGAGCACTCGAGATGATAG-3’; |
| Sh2 | 5’-GGTGGCATGTTGTCCTCAATC-3’; |
| Sh3 | 5’-GGGAGACCAAAGTTATCAAGG-3’; |
| Scr | 5’-GGTTAAGTCGCCCTCGCTC-3’ |

**The siRNAs sequences**

| Name | sequence |
| --- | --- |
| KDM3A | 5’-CCGACGTTACCAAGAAGGATCTGAA-3’ |
| KDM3B | 5’-CCTAGCGATCTTTGTAGAATTTGAT-3’ |
| KDM4A | 5’-CAGCTGCCTTGGATCTTTCTGTGAA-3’ |
| KDM4B  KDM5B  JMJD6  PHF2 | 5’-TCGCCCAACCATGGAAGAATTTAAA-3’  5’-CAGTTGTGTGGCGGTACCCAGTATT-3’  5’-GAGGATAACGATGGCTACTCAGTGA-3’  5’-CAGGTCGACAAATGCTACAAGTGCA-3’ |

**The qPCR primers**

| Name | Forward 5’-3’ | Reverse5’-3’ |
| --- | --- | --- |
| KDM3A | ACCTGCAGTTATTCTTCAGC | TAATGCCAGTCCTATGCCAT |
| KDM3B | TGTTCCCTGGGGACTCCTCT | GGGCACTACAGTACAGCTGG |
| KDM4A | CCTCACTGCGCTGTCTGTAT | CCAGTCGAAGTGAAGCACAT |
| KDM4B | CGGGTTCTATCTTTGTTTCTCTCACCCG | AAGGAAGCCTCTGGAACACCTG |
| KDM5B | AAGGAAGCCTCTGGAACACCTG | GCAGAGTCTGGGAATTCACA |
| JMJD6 | AGGTGGATCACTTGAGGTCA | CACCACACCTGGCTAATTTT |
| PHF2 | TCGGCACTTCTCTGTTCTCCC | AAATCCAGCCCCTCCGTGTC |
| β-actin | AGAGGGAAATCGTGCGTGAC | CAATAGTGATGACCTGGCCGT |
| ITGA8 | GCTGCTGGGGAGTTTACTGG | GATGCCATCTGTTCTCCCGTG |
| G0S2 | CGCCGTGCCACTAAGGTC | GCACACAGTCTCCATCAGGC |
| CDKN1C | TGACCTCCTTCAGCGAGTG | TCGGGACTTCTGCGTCATC |
| PSAT1 | GGCCAGTTCAGTGCTGTCC | GCTCCTGTCACCACATAGTCA |
| PDE1C | GTGACTGAGCAACCATAGTGGAC | TCGCTGGACAATGTCACTCCTG |
| GDF15 | CTCCAGATTCCGAGAGTTGC | AGAGATACGCAGGTGCAGGT |
| VCAM1 | GCGGAGACAGGAGACACAGTACTAA | GAGCACGAGAAGCTCAGGAGAA |
| MT1 | AGTCTCTCCTCGGCTTGC | ACATCTGGGAGAAAGGTTGTC |

**The primers used for ChiP**

| Name | Forward 5’-3’ | Reverse5’-3’ |
| --- | --- | --- |
| PSAT1- primer1 | GGGCCACCTTCTTCTGGTTT | GGGAAACGAGTGAGCTGGAA |
| PSAT1- primer2 | AGCGGATGCATGAATGGACA | CACTGGTGTAAGGCGTAGGG |
| PDE1C-primer1 | AGGTGTGGTGTTCATTCCCG | GATTCGGGGGCCCCATTTAT |
| PDE1C-primer2 | TGGACTTTGTCAGTGGGTGG | TACAGTATGGGGGTGGGACC |
| PDE1C-primer3 | CTGCCATTTACTGCTTGCCA | TCTACCCAGCTTGGCAGTTG |
