# Supplementary figures and images for: Histone demethylase JMJD6 regulates cellular migration and proliferation in adipose-derived mesenchymal stem cells
Source: Stem Cell Res Ther. 2018 Aug 9;9:212. doi: 10.1186/s13287-018-0949-3 (PMC6085710; doi:10.1186/s13287-018-0949-3)

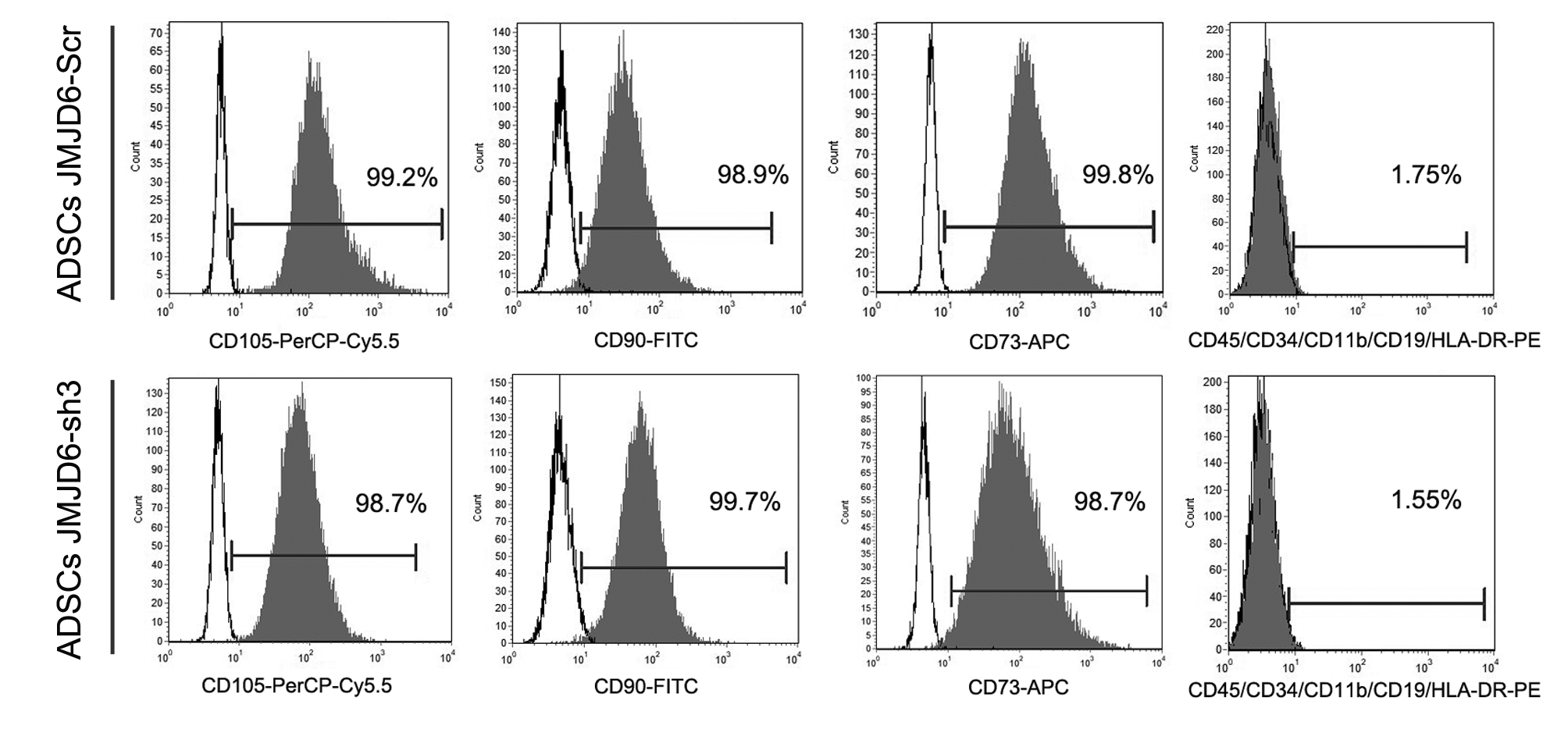

Supplement: Supplementary file 2 — Figure S1. Effects of JMJD6 loss on cell surface marker expression in ADSCs. The graphs show the ADSCs surface antigen phenotypes. Positive expression markers: CD90-FITC, CD73-APC, and CD105-PerCPCy5.5. Negative expression markers: (CD45/CD34/CD11b/CD19/ HLA-DR PE). (TIF 407 kb) [file 13287_2018_949_MOESM2_ESM.tif]

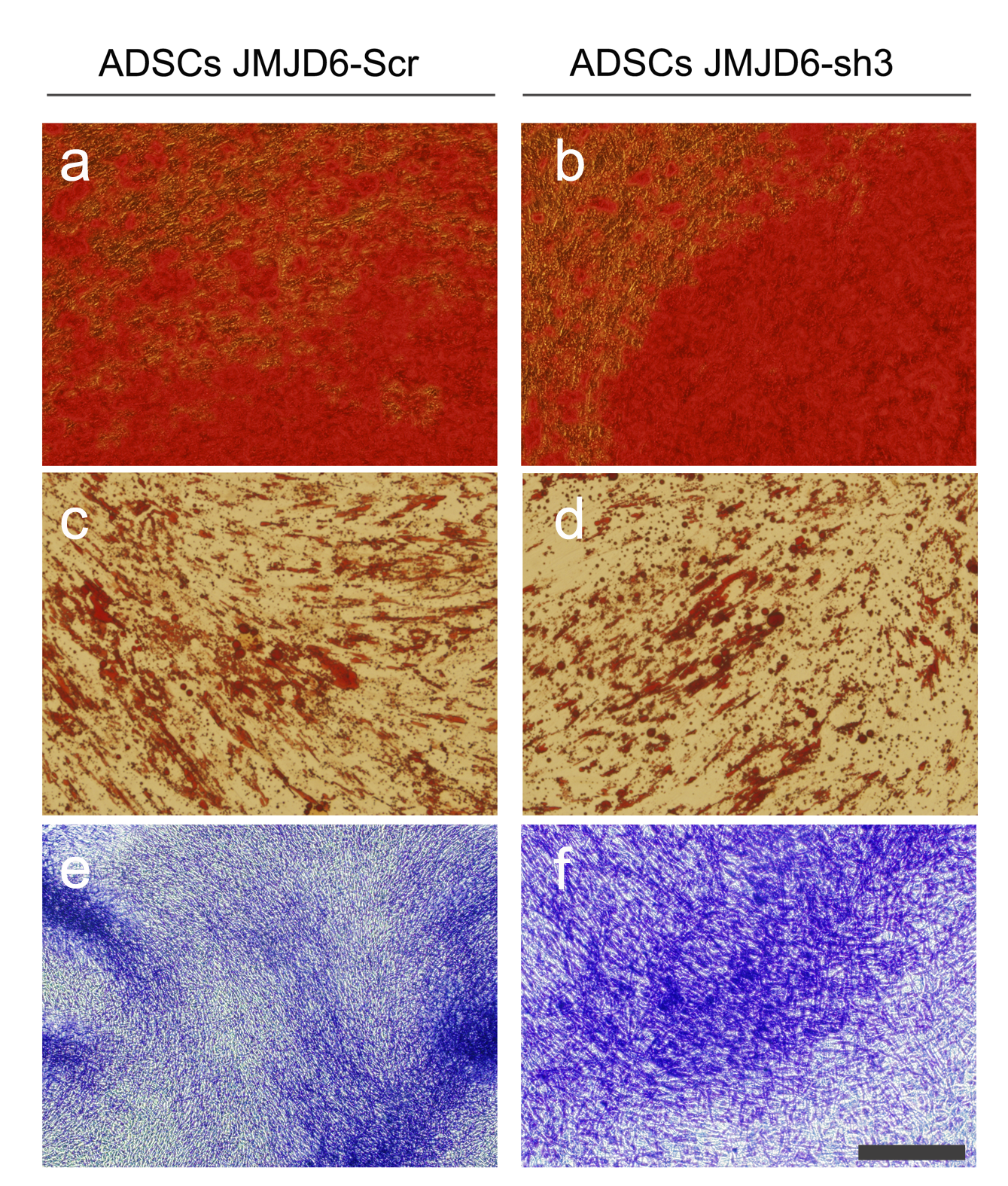

Supplement: Supplementary file 3 — Figure S2. The osteogenic, adipogenic, and chondrogenic differentiation of ADSCs. After ADSCs were cultured in vitro for 16 days, osteogenic differentiation was analyzed by Alizarin Red staining (a, b). Adipogenic differentiation was measured by Oil Red O staining (c, d) and chondrogenic differentiation capacity was examined by Toluidine Blue staining (e, f). Scale bar = 200 μm, magnification = ×50. (TIF 9808 kb) [file 13287_2018_949_MOESM3_ESM.tif]
